# The prevalence of *Helicobacter pylori* infection in inflammatory bowel disease in China: A case-control study

**DOI:** 10.1371/journal.pone.0248427

**Published:** 2021-03-12

**Authors:** Zhao-Hui Ding, Xiao-Ping Xu, Tian-Rong Wang, Xiao Liang, Zhi-Hua Ran, Hong Lu

**Affiliations:** 1 Division of Gastroenterology and Hepatology, Shanghai Institute of Digestive Disease, Key Laboratory of Gastroenterology & Hepatology, Ministry of Health, RenJi Hospital, School of Medicine, Shanghai Jiao Tong University, Shanghai, China; 2 Clinical Biochemistry Laboratory, RenJi Hospital, School of Medicine, Shanghai Jiao Tong University, Shanghai, China; Oita University Faculty of Medicine, JAPAN

## Abstract

**Background & aims:**

*Helicobacter pylori (H*. *pylori*) infection remains high in China though the incidence of inflammatory bowel disease (IBD) has increased. Our aim was to investigate the relationship between the prevalence of *H*. *pylori* and inflammatory bowel disease.

**Methods:**

Hospitalized IBD patients including Crohn’s disease (CD) and ulcerative colitis (UC) who had tested *H*. *pylori* antibody were enrolled. Controls were chose from age- and sex- matched healthy physical examination people who had *H*. *pylori* antibody test in a 1:2 fashion (IBD patients:controls). IBD medical history was recorded. All patients were typed by the Montreal classification. Mayo Clinic score and the Harvey-Bradshaw Severity Index were used to evaluate their disease activity. Patients and controls that had *H*. *pylori* eradication therapy before were excluded.

**Results:**

Two hundred and sixty IBD patients including 213 CD patients and 47 UC patients, and 520 controls were involved in this study. The prevalence of *H*. *pylori* infection in IBD patients (9.6%, 25/260) and IBD newly diagnosed patients (12.1%, 8/66), as well as CD patients (8.9%, 19/213) including CD newly diagnosed patients (10.6%, 5/47) and UC patients (12.8%, 6/47) was significantly lower than controls (29.8%, 155/520) (*p* = 2.796*10^−10^, 0.007, 5.723*10^−9^, 0.016, 0.014), while there was no statistically difference between UC newly diagnosed patients and the controls, and IBD patients with different disease type, disease activity and treatment history.

**Conclusions:**

*H*. *pylori* infection had a negative association with IBD, especially CD.

## Introduction

*Helicobacter pylori* (*H*. *pylori*), a common Gram-negative, gastric mucosa colonized microaerobic bacteria, classified as a Class I carcinogen by the World Health Organization, can cause infectious disease and is associated with peptic ulcers, gastric cancer and MALT lymphoma. It has also been reported to be negatively connected to asthma and esophageal adenocarcinoma [1]. In recent years, the prevalence of *H*. *pylori* in China has gradually declined, but still at a high level [2, 3]. Meanwhile, with economic development and the westernization of lifestyle, the incidence of inflammatory bowel disease (IBD) is climbing [4] including Crohn’s disease (CD) and ulcerative colitis (UC). Some epidemiology studies from Western believed that *H*. *pylori* infection was a protective factor for IBD [5] and some animal studies showed the protective effects of *H*. *pylori* in colitis [6]. Other researchers such as Bell *et al*. didn’t identify any Helicobactor species in the colonic biopsies of IBD patients by multiple polymerase chain reactions (PCR) and indicated that it was not related to the occurrence of IBD [7]. However, the data on the relationship between these two diseases in China is still insufficient. Our aim is to study the relationship between *H*. *pylori* status and IBD patients in China.

## Methods

### Subjects

Study population consisted of 260 hospitalized IBD patients, who had *H*. *pylori* serology test during their hospitalization in the Department of Gastroenterology of Renji Hospital Affiliated to Shanghai Jiao Tong University School of Medicine, between June 2019 and July 2020. Hospitalized IBD patients included newly diagnosed patients (naïve patients), patients with IBD flare, day hospitalization patients for IV infliximab or adalimumab, and regular reexamination patients. The diagnosis of CD and UC was based on the 2018 Chinese Consensus on diagnosis and treatment of inflammatory bowel disease [8]. Those who got their diagnosis first time during the hospitalization were defined as naïve patients or newly diagnosed patients. Five hundred and twenty sex- and age-matched (±5 years) controls were selected in a 1:2 fashion (IBD patients:controls) from healthy people who came to have physical examination in the same hospital between the same period. Patients and controls who had *H*. *pylori* eradication therapy records before were excluded. Previous medical history including age of onset and IBD treatment (infliximab, adalimumab, 5-aminosalicylic acid, sulfasalazine, glucocorticoids, azathioprine, tacrolimus) was obtained from medical records. CD and UC patients were classified according to The Montreal classification [9] and the disease activity was also evaluated by the Harvey-Bradshaw Severity Index [10] and the Mayo Clinic score [11]. All of the data including *H*. *pylori* serology test results were collected from medical records and were fully anonymized before, during and after our access. The Ethics Committee of Renji Hospital Affiliated to Shanghai Jiao Tong University School of Medicine approved this retrospective study (KY2020-139) and waived the requirement for informed consent.

### *H*. *pylori* status

Serology test was used as a reference basis for *H*. *pylori* infection analysis. Peripheral venous serum samples of patients and controls were tested by *H*. *pylori* antibody kit (Latex Immunoturbidimetric method) (Beijing Wan Tai DRD Co., LTD). Reference range: negative: <22 AU/ml; positive: ≥22 AU/ml.

### Sample size calculation

According to previous study, the *H*. *pylori* prevalence in China is 55.8% [12], and the odds ratio (OR) between IBD and *H*. *pylori* infection is 0.43 [13]. The minimum sample sizes are 139 IBD patients and 278 controls, with a significance of 0.005, a power of 90% and an unbalanced sample ratio 1:2 (IBD patients:controls).

### Statistical analysis

Categorical variables were analyzed by Chi Square/Fisher’s exact test and continuous variables by T-test/Mann Whitney test. A statistical p value of 0.05 (two sided) was considered significant for all comparisons. Odds ratios (OR) with corresponding 95% confidence intervals (CIs) were calculated for analyzing the association between *H*. *pylori* infection and IBD onset. All computations were performed with the SPSS 26.00 statistical software.

## Results

Two hundred and sixty seven patients (213 CD and 47 UC) and 520 sex- and age-matched controls were enrolled in this study. The detailed demographic and clinical characteristics are shown in Table 1.

**Table 1:** Demographic and clinical features.

		CD	UC	IBD	Controls
		(n = 213)	(n = 47)	(n = 260)	(n = 520)
Gender, n (%)	Male	148 (69.5)	21 (44.7)	169 (65.0)	338 (65.0)
	Female	65 (30.5)	26 (55.3)	91 (35.0)	182 (35.0)
Age (Mean±SD), y		33.4±11.6	41.8±15.1	34.9±12.7	36.0±11.6
Age at diagnosis (Mean±SD), y		28.2±10.7	36.7±14.5		
Disease duration (Mean±SD), y		5.2±4.5	5.1±6.9		
Naïve patients*, n (%)		47 (22.1)	19 (40.4)	66 (25.4)	

CD: Crohn’s disease, UC: ulcerative colitis;

*: Newly diagnosed patients.

### *H*. *pylori* status

Overall, only 25 IBD patients (25/260, 9.6%, 19 for CD patients and 6 for UC patients) had positive *H*. *pylori* serology, which were significantly lower than controls (155/520, 29.8%) (*p* = 2.796*10^−10^). The *H*. *pylori* prevalence of CD (19/207, 9.2%) and UC (6/47, 12.8%) patients was also separately lower than their controls (125/426, 29.3%; 20/94, 31.9%) (*p* = 5.723*10^−9^; 0.014).

In the subgroups analysis of IBD naive patients, the *H*. *pylori* prevalence (8/66, 12.1%) was slightly higher than all IBD patients (25/260, 9.6%) (*p* = 0.547), but still lower than the control group (39/132, 29.5%) (*p* = 0.007). And CD naive patients (5/47, 10.6%) were also significantly lower than their controls (27/94, 28.7%, *p* = 0.016) while the UC ones (3/19, 15.8%) has no statistically difference with their controls (12/38, 31.6%, *p* = 0.202). (Table 2).

**Table 2:** *H*. *pylori* status in IBD patients and controls.

	*H*. *pylori* prevalence	X^2^	*p*	OR (CI 95%)
CD	8.9% (19/213)	33.927	5.723*10^−9^	0.236
controls	29.3% (125/426)			(0.141–0.395)
UC	12.8% (6/47)	6.043	0.014	0.312
controls	31.9% (30/94)			(0.120–0.816)
IBD	9.6% (25/260)	39.813	2.796*10^−10^	0.251
controls	29.8% (155/520)			(0.159–0.394)
CD naive*	10.6% (5/47)	5.841	0.016	0.296
Controls	28.7% (27/94)			(0.106–0.827)
UC naive*	15.8% (3/19)	1.629	0.202	0.406
Controls	31.6% (12/38)			(0.099–1.664)
IBD naive*	12.1% (8/66)	7.379	0.007	0.329
controls	29.5% (39/132)			(0.144–0.753)

CD: Crohn’s disease, UC: ulcerative colitis; IBD, inflammatory bowel disease; OR: odds ratio; CI: confidence interval.

*: Newly diagnosed patients.

### *H*. *pylori* status with IBD classification and activity

There is no statistically difference of the *H*. *pylori* infection rate in CD and UC patients with different ages, lesion parts, disease behaviors, perianal lesions and activity levels. (S1 and S2 Tables).

### *H*. *pylori* status with IBD treatment history

There is no significant difference of the prevalence of *H*. *pylori* between IBD patients who had treatment history of infliximab or adalimumab, 5-aminosalicylic acid, sulfasalazine, glucocorticoids, azathioprine, tacrolimus, and that of patients who hadn’t used such medication. (S3 and S4 Tables).

## Discussion

The etiology and mechanism of inflammatory bowel disease is currently not fully explicit, but some specific factors are found related to IBD, including host genetic factors, environments and diets changing, intestinal immune system, and changes in intestinal microbiota [14]. Over the past two decades, with the economic development and western lifestyle adopted in China, the incidence of IBD is also reported rapidly increasing [4].

Western researchers have conducted a lot of correlation study between *H*. *pylori* prevalence and IBD. A meta-analysis, involved 40 literatures, enrolled 80789 patients from 17 countries, found that regardless of race, age, and test method, *H*. *pylori* infection was significantly reduced in IBD patients [13]. Teplar *et al*. used a random effects model and showed that the incidence of IBD, especially CD was significantly reduced in CagA seropositive patients, while the exposure of CagA seronegative *H*. *pylori* was not significantly associated with the incidence of IBD [15]. Our study suggests that the prevalence of *H*. *pylori* infection in the IBD group, including CD and UC patients, is significantly lower than that in the general health population, which is consistent with most research results.

The low *H*. *pylori* infection rate of IBD patients may also be relevant to the improvement of socioeconomic conditions and special host genetic factors such as ATG16L1 [16] and the widespread use of antibacterial drugs. Yang *et al*. suggested that in IBD patients who have received tetracycline and quinolone antibacterial drugs for more than 7 days, their *H*. *pylori* infection rate was significantly lower than that of patients without a history of treatment [17]. Our study did not collect the history of antimicrobial drugs in patients. Though there is no statistical difference, we found that naïve IBD patients have a relatively higher *H*. *pylori* prevalence among all the patients, and with the aggravation of CD disease behavior (structuring and penetrating), the infection rate relatively decreased. The reason might be more chance to use antibiotics in the progression and exacerbation of the disease.

In addition, Frost et al. analyzed fecal microbiota in 212 *H*. *pylori* positive patients and controls, and found *H*. *pylori* related to increased fecal microbes diversity [18]. Other studies [19, 20] including Sonnenberg et al., who studied biopsies from 302,061 upper and lower endoscopy patients (13,943 IBD), found that weakened gastric acid barrier may protect against IBD development [19]. Therefore, *H*. *pylori* may regulate lower digestive tract microbiota through the change of gastric acid secretion [21] and relate to the incidence of IBD. Furthermore, *H*. *pylori* chromosomal DNA can prevent colitis by inhibiting the production of type I interferon and IL-12 in the mouse model [22], while type I T helper lymphocytes and Th17-related cytokines (include IL-12) are selectively activated in CD [23]. The decrease in *H*. *pylori* infection may contribute the pathogeneses of IBD, especially CD.

## Conclusions

Our study shows that the prevalence of *H*. *pylori* infection in IBD patients, especially CD, is lower than general population. *H*. *pylori* infection might be a protective factor for CD, or it might be two outcomes influenced by some common factors, which still need further study.

## Supporting information

S1 Table Disease classification, activity and *H*. *pylori* status in CD patients. (DOCX)

S2 Table Disease classification, activity and *H*. *pylori* status in UC patients. (DOCX)

S3 Table *H*. *pylori* status with CD treatment history. (DOCX)

S4 Table *H*. *pylori* status with UC treatment history. (DOCX)

S1 File Data. (XLSX)

S2 File STROBE checklist. (DOCX)
