# Supplementary material for: The prevalence of Helicobacter pylori infection in inflammatory bowel disease in China: A case-control study
Source: PLoS One. 2021 Mar 12;16(3):e0248427. doi: 10.1371/journal.pone.0248427 (PMC7954320; doi:10.1371/journal.pone.0248427)
Supplement: S1 Table — (DOCX) [file pone.0248427.s001.docx]

**S1 Table. Disease classification, activity and *H. pylori* status in CD patients**

|  |  | *H. pylori* prevalence | | X^2^ | p |
| --- | --- | --- | --- | --- | --- |
| A^1^ | 1 | 14.3% (2/14) | 1.210 | | 0.541 |
|  | 2 | 8.2% (14/170) |  | |  |
|  | 3 | 10.3% (3/29) |  | |  |
| L^1^ | 1 | 10.8% (7/65) | 0.749 | | 0.884 |
|  | 2 | 6.7% (1/15) |  | |  |
|  | 3 | 8.1% (10/123) |  | |  |
|  | 4 | 10.0% (1/10) |  | |  |
| B^1^ | 1 | 10.5% (11/105) | 1.635 | | 0.465 |
|  | 2 | 9.8% (6/61) |  | |  |
|  | 3 | 4.3% (2/47) |  | |  |
| P^1^ | 0 | 7.1% (5/70) | 0.405 | | 0.524 |
|  | 1 | 9.8% (14/143) |  | |  |
| HBSI^2^ | Remission | 8.3% (13/157) | 0.697 | | 0.825 |
|  | Mild | 11.6% (5/43) |  | |  |
|  | Moderate | 7.7% (1/13) |  | |  |

1 Based on Montreal classification for Crohn's disease, A: Age of diagnosis; L: Location; B: Behavior; p: perianal disease modifier,

2 HBSI: Harvey-Bradshaw Severity Index, remission (score＜5 points), mild activity (score of 5 to 7 points), moderate activity (score of 8 to 16 points), severe activity (score >16 points).
