# Supplementary material for: The prevalence of Helicobacter pylori infection in inflammatory bowel disease in China: A case-control study
Source: PLoS One. 2021 Mar 12;16(3):e0248427. doi: 10.1371/journal.pone.0248427 (PMC7954320; doi:10.1371/journal.pone.0248427)
Supplement: S2 Table — (DOCX) [file pone.0248427.s002.docx]

**S2 Table. Disease classification, activity and *H. pylori* status in UC patients**

|  |  | *H. pylori* prevalence | X^2^ | p |
| --- | --- | --- | --- | --- |
| E^1^ | 1 | 0.0% (0/5) | 1.682 | 0.408 |
|  | 2 | 23.1% (3/13) |  |  |
|  | 3 | 10.3% (3/29) |  |  |
| Mayo Clinic score^2^ | Remission | 0% (0/5) | 2.042 | 0.393 |
|  | Mild | 25% (3/12) |  |  |
|  | Moderate | 10% (3/30) |  |  |

1 Based on Montreal classification of Extent of ulcerative colitis

2 Mayo Clinic score: remission (score < 3 points), mild activity (score of 3 to 5 points, or one subscore = 2), moderate activity (score of 6 to 10 points), severe activity (score >10 points).
