# Supplementary material for: The prevalence of Helicobacter pylori infection in inflammatory bowel disease in China: A case-control study
Source: PLoS One. 2021 Mar 12;16(3):e0248427. doi: 10.1371/journal.pone.0248427 (PMC7954320; doi:10.1371/journal.pone.0248427)
Supplement: S3 Table — (DOCX) [file pone.0248427.s003.docx]

**S3 Table. *H. pylori* status with CD treatment history**

|  | Used | Not used | X^2^ | p |
| --- | --- | --- | --- | --- |
| Infliximab or Adalimumab | 10.8% (13/121) | 6.5% (6/92) | 1.147 | 0.284 |
| 5-aminosalicylic acid | 10.3% (12/117) | 7.3% (7/96) | 0.57 | 0.450 |
| Sulfasalazine | 0.0% (0/16) | 9.6% (19/197) | 0.715 | 0.398 |
| Glucocorticoids | 5.7% (5/88) | 11.2% (14/125) | 1.936 | 0.164 |
| Azathioprine | 9.4% (9/96) | 8.5% (10/117) | 0.044 | 0.833 |
| Tacrolimus | 0.0% (0/13) | 9.5% (19/200) | 0.439 | 0.508 |
