# Supplementary material for: The prevalence of Helicobacter pylori infection in inflammatory bowel disease in China: A case-control study
Source: PLoS One. 2021 Mar 12;16(3):e0248427. doi: 10.1371/journal.pone.0248427 (PMC7954320; doi:10.1371/journal.pone.0248427)
Supplement: S4 Table — (DOCX) [file pone.0248427.s004.docx]

**S4 Table. *H. pylori* status with UC treatment history**

|  | Used | Not used | X^2^ | p |
| --- | --- | --- | --- | --- |
| Infliximab or Adalimumab | 14.3% (1/7) | 12.5% (5/40) |  | 1 |
| 5-aminosalicylic acid | 14.3% (6/42) | 0% (0/5) |  | 1 |
| Sulfasalazine | 0% (0/9) | 15.8% (6/38) | 0.520 | 0.471 |
| Glucocorticoids | 8.3% (2/24) | 17.4% (4/23) | 0.243 | 0.622 |
| Azathioprine | 16.7% (1/6) | 12.2% (5/41) |  | 1 |
| Tacrolimus | 50% (1/2) | 11.1% (5/45) |  | 0.241 |
