# Supplementary material for: The prevalence of Helicobacter pylori infection in inflammatory bowel disease in China: A case-control study
Source: PLoS One. 2021 Mar 12;16(3):e0248427. doi: 10.1371/journal.pone.0248427 (PMC7954320; doi:10.1371/journal.pone.0248427)
Supplement: S2 File — (DOCX) [file pone.0248427.s006.docx]

STROBE Statement—checklist of items that should be included in reports of observational studies

|  | Item No. | Recommendation | Page  No. | Relevant text from manuscript |
| --- | --- | --- | --- | --- |
| **Title and abstract** | 1 | (*a*) Indicate the study’s design with a commonly used term in the title or the abstract | 1 | The prevalence of Helicobacter pylori infection in inflammatory bowel disease in China: A case-control study |
|  |  | (*b*) Provide in the abstract an informative and balanced summary of what was done and what was found | 2-3 | **Methods**  Hospitalized IBD patients including…  **Results**  Two hundred and sixty IBD patients…  **Conclusions**  *H. pylori* infection had a negative… |
| Introduction | | | |  |
| Background/rationale | 2 | Explain the scientific background and rationale for the investigation being reported | 4 | *Helicobacter pylori* (*H. pylori*), a common Gram-negative…Some studies from Western believed…However, the data on the relationship between these two diseases in China is still insufficient… |
| Objectives | 3 | State specific objectives, including any prespecified hypotheses | 4 | Our aim is to study the relationship between *H. pylori* status and IBD patients in China. |
| Methods | | | |  |
| Study design | 4 | Present key elements of study design early in the paper | 4 | Study population consisted of 260 hospitalized IBD patients…Five hundred and twenty sex- and age-matched (±5 years) controls… |
| Setting | 5 | Describe the setting, locations, and relevant dates, including periods of recruitment, exposure, follow-up, and data collection | 4-5 | at the Department of Gastroenterology of Renji Hospital Affiliated to Shanghai Jiao Tong University School of Medicine, between July 2019 and January 2020 |
| Participants | 6 | (*a*) *Cohort study*—Give the eligibility criteria, and the sources and methods of selection of participants. Describe methods of follow-up  *Case-control study*—Give the eligibility criteria, and the sources and methods of case ascertainment and control selection. Give the rationale for the choice of cases and controls  *Cross-sectional study*—Give the eligibility criteria, and the sources and methods of selection of participants | 4-5 | …consisted of 260 hospitalized IBD patients…controls were selected…from healthy physical examination people between the same period. The diagnosis of CD and UC was based on the 2018 Chinese Consensus. Patients who had *H. pylori* eradication therapy before were excluded. |
|  |  | (*b*) *Cohort study*—For matched studies, give matching criteria and number of exposed and unexposed  *Case-control study*—For matched studies, give matching criteria and the number of controls per case | 4 | Five hundred and twenty sex- and age-matched (±5 years) controls were selected in a 1:2 fashion (IBD patients:controls) from healthy people |
| Variables | 7 | Clearly define all outcomes, exposures, predictors, potential confounders, and effect modifiers. Give diagnostic criteria, if applicable | 4-5 | The diagnosis of CD and UC was based on the 2018 Chinese Consensus on diagnosis and treatment of inflammatory bowel disease [7] |
| Data sources/ measurement | 8* | For each variable of interest, give sources of data and details of methods of assessment (measurement). Describe comparability of assessment methods if there is more than one group | 5 | …age of onset and IBD treatment…CD and UC patients were classified according to The Montreal classification [8] and the disease activity was also evaluated by the Mayo Clinic score[9] and the Harvey-Bradshaw Severity Index[10]. Serology test was used as a reference basis…Reference range: negative: <22 AU/ml; positive: ≥22 AU/ml. |
| Bias | 9 | Describe any efforts to address potential sources of bias | 4 | sex- and age-matched (±5 years) controls were selected in a 1:2 fashion. Patients who had *H. pylori* eradication therapy before were excluded. |
| Study size | 10 | Explain how the study size was arrived at | 5 | …The minimum sample sizes are 139 IBD patients and 278 controls with a significance of 0.005, a power of 90%… |

Continued on next page

| Quantitative variables | 11 | Explain how quantitative variables were handled in the analyses. If applicable, describe which groupings were chosen and why | 6 | continuous variables by T-test/Mann Whitney test |
| --- | --- | --- | --- | --- |
| Statistical methods | 12 | (*a*) Describe all statistical methods, including those used to control for confounding | 5 | Categorical variables were analyzed by Chi Square/Fisher’s exact test |
|  |  | (*b*) Describe any methods used to examine subgroups and interactions | 6 | Categorical variables were analyzed by Chi Square/Fisher’s exact test… |
|  |  | (*c*) Explain how missing data were addressed |  | N/A |
|  |  | (*d*) *Cohort study*—If applicable, explain how loss to follow-up was addressed  *Case-control study*—If applicable, explain how matching of cases and controls was addressed  *Cross-sectional study*—If applicable, describe analytical methods taking account of sampling strategy | 4 | sex- and age-matched (±5 years) controls were selected in a 1:2 fashion (IBD patients:controls)… |
|  |  | (*e*) Describe any sensitivity analyses |  | N/A |
| Results | | | | |
| Participants | 13* | (a) Report numbers of individuals at each stage of study—eg numbers potentially eligible, examined for eligibility, confirmed eligible, included in the study, completing follow-up, and analysed | 4-5 | …consisted of 260 hospitalized IBD patients…Patients who had *H. pylori* eradication therapy records before were excluded… |
|  |  | (b) Give reasons for non-participation at each stage |  | N/A |
|  |  | (c) Consider use of a flow diagram |  | N/A |
| Descriptive data | 14* | (a) Give characteristics of study participants (eg demographic, clinical, social) and information on exposures and potential confounders | 6 | Table1. Demographic and clinical features |
|  |  | (b) Indicate number of participants with missing data for each variable of interest |  | N/A |
|  |  | (c) *Cohort study*—Summarise follow-up time (eg, average and total amount) |  | N/A |
| Outcome data | 15* | *Cohort study*—Report numbers of outcome events or summary measures over time |  | N/A |
|  |  | *Case-control study—*Report numbers in each exposure category, or summary measures of exposure | 6 | Overall, only 25 IBD patients (25/260, 9.6%, 19 for CD patients and 6 for UC patients) had positive *H. pylori* serology… |
|  |  | *Cross-sectional study—*Report numbers of outcome events or summary measures |  | N/A |
| Main results | 16 | (*a*) Give unadjusted estimates and, if applicable, confounder-adjusted estimates and their precision (eg, 95% confidence interval). Make clear which confounders were adjusted for and why they were includeda | 7 | Table 2. *H. pylori* status in IBD patients and controls |
|  |  | (*b*) Report category boundaries when continuous variables were categorized |  | N/A |
|  |  | (*c*) If relevant, consider translating estimates of relative risk into absolute risk for a meaningful time period |  | N/A |

Continued on next page

| Other analyses | 17 | Report other analyses done—eg analyses of subgroups and interactions, and sensitivity analyses | 6-9 | In the subgroups analysis of IBD naive patients…The infection rate decreases with the aggravation of CD…between IBD patients who had treatment history of infliximab or adalimumab… |
| --- | --- | --- | --- | --- |
| Discussion | | | | |
| Key results | 18 | Summarise key results with reference to study objectives | 10 | Our study suggests that the prevalence of *H. pylori* infection in the IBD group, including CD and UC patients respectively, is significantly lower than… |
| Limitations | 19 | Discuss limitations of the study, taking into account sources of potential bias or imprecision. Discuss both direction and magnitude of any potential bias | 10 | Our study did not collect the history of antimicrobial drugs in patients |
| Interpretation | 20 | Give a cautious overall interpretation of results considering objectives, limitations, multiplicity of analyses, results from similar studies, and other relevant evidence | 11 | **Conclusions** Our study shows that the prevalence of *H. pylori* infection in IBD patients, especially CD, is lower than general population. |
| Generalisability | 21 | Discuss the generalisability (external validity) of the study results | 10 | the prevalence of H. pylori infection in the IBD group, including CD and UC patients, is significantly lower than that in the general health population, which is consistent with most research results. |
| Other information | |  | | |
| Funding | 22 | Give the source of funding and the role of the funders for the present study and, if applicable, for the original study on which the present article is based | 11 | We would like to acknowledge National Natural Science Foundation of China (81970497) for funding our study. The funders had no role…of the manuscript. |

*Give information separately for cases and controls in case-control studies and, if applicable, for exposed and unexposed groups in cohort and cross-sectional studies.

**Note:** An Explanation and Elaboration article discusses each checklist item and gives methodological background and published examples of transparent reporting. The STROBE checklist is best used in conjunction with this article (freely available on the Web sites of PLoS Medicine at http://www.plosmedicine.org/, Annals of Internal Medicine at http://www.annals.org/, and Epidemiology at http://www.epidem.com/). Information on the STROBE Initiative is available at www.strobe-statement.org.
